# Evaluation of HIV Late Presentation Trends: A Cross-Sectional Analysis from a Leading Ecuadorian Public Hospital

**DOI:** 10.3390/pathogens14060598

**Published:** 2025-06-18

**Authors:** Adriana D. Suarez-Vizcaino, Nicole C. Bustamante-Pancho, Juan S. Izquierdo-Condoy, Hugo Pereira-Olmos, I. Alberto Castillo, Esteban Ortiz-Prado

**Affiliations:** 1 Ministerio de Salud Pública del Ecuador, Quito 170702, Ecuador; 2 One Health Research Group, Faculty of Medicine, Universidad de las Américas, Quito 170137, Ecuador; 3 Public Health Institute, Faculty of Medicine, Pontificia Universidad Catolica del Ecuador, Quito 170146, Ecuador; 4 Internal Medicine Postgraduate Program, Falcultad de Medicina, Pontifica Universidad Catolica del Ecuador, Quito 170146, Ecuador

**Keywords:** HIV, AIDS, late presentation, diagnosis, CD4 T lymphocytes, screening

## Abstract

The global impact of HIV is especially significant when diagnoses are made in advanced stages. While strategies exist to mitigate late presentations, Ecuador’s 2018–2022 strategic plan has not yet been evaluated. This study assesses the prevalence and implications of late and advanced HIV presentations in Ecuador, using data from a reference hospital in Quito. A cross-sectional analysis of 436 medical records of people living with HIV from the “Hospital de Especialidades Eugenio Espejo” was conducted between November 2015 and February 2020. The data were divided into “Pre-Plan” and “Post-Plan” periods for comparative analysis. The mean CD4 T count showed a non-statistically significant increase in the post-plan period (January 2018–February 2020). Notably, 65.1% of patients presented late, and 39.4% had advanced disease. Demographic data indicated that 89.9% were men, and 54.1% were under 30 years of age. No characteristics were identified that were associated with advanced late presentation of HIV infection. Sexual orientation data revealed that 69.1% identified as homosexual or bisexual. A predominance of late and advanced presenters was identified in the post-plan period, associated with being employed (*p* < 0.05) and being drug users (*p* < 0.001). There was also a greater incidence of late presenters among immigrants in the post-plan period (*p* = 0.045). Despite the implementation of Ecuador’s 2018–2022 strategic plan for HIV, substantial challenges in reducing late presentations remain. This study suggests that early diagnoses have not significantly improved. Employed patients and drug users were more likely to present late, with drug users also accounting for many advanced cases. This study highlights the need for more focused and targeted strategies to supplement the existing plan.

## 1. Introduction

The human immunodeficiency virus (HIV) is responsible for an infection affecting over 39 million people worldwide. In 2022 alone, more than 1.3 million people were newly infected worldwide [1]. In Ecuador, it is estimated that by 2020, 45,056 people were living with HIV (PLWH). The most affected age group is 15 to 49 years old, with a higher incidence in men. The epidemic in Ecuador is concentrated and mainly affects key population groups (CPG), especially men who have sex with men (MSM), with rates of 16.5% in Quito and 11.2% in Guayaquil the main urban centers, and transfeminine women, with rates of 34.8% in Quito and 20.7% in Guayaquil [2].

The introduction of antiretroviral therapy (ART) has transformed HIV from a fatal disease into a manageable chronic condition. ART effectively suppresses viral replication, prevents AIDS-related complications, reduces HIV transmission, and significantly improves life expectancy and quality of life among PLWH [3].

Although HIV treatment has advanced, the infection can still progress to a state of immunosuppression characterized by a marked reduction in CD4 T lymphocytes (CD4 T). This condition can be managed if diagnosed early and treated adequately [4,5]. A significant concern is patients who receive medical attention only after the infection has progressed, termed late infection. Late presentation is defined as CD4 T count <350 to 200 cells/mm^3^. Advanced disease was defined as CD4 T count <200 cells/mm^3^ or the presence of an acquired immune deficiency syndrome (AIDS)-defining illness [6]. Consequences for these patients include more rapid disease progression, increased risk of non-infectious morbidity, delayed immune system recovery even after ART, and a 3.5 times higher rate of developing opportunistic infections compared to those without late presentation. These patients also face lower survival rates and higher medical costs [7,8,9]. Additionally, some patients may be diagnosed even later, classified as having advanced disease with an initial CD4 T count of less than 200 cells/mm^3^ or the presence of an AIDS-defining illness, leading to a markedly worse prognosis and even lower survival rates [6]. 

Early HIV testing is therefore essential. Early diagnosis enables timely initiation of ART, halts viral replication before significant immunosuppression occurs, reduces HIV transmission in the community, and improves individual outcomes. Moreover, early detection has epidemiological benefits, as it decreases the community viral load and curbs further spread [10].

Research indicates that the number of late presenters of HIV is associated with the viral load in their community [11], making it a concern in various countries. Studies in England, China, the Netherlands, Switzerland, the United States, and Scotland have identified risk factors related to late presentation, including male gender; older age; specific ethnicities such as African, Asian, and Hispanic; lower educational levels; marital statuses such as married, divorced, or widowed; unemployment; migration; specific transmission routes such as sexual transmission or injection drug use; heterosexual orientation; and lack of health insurance [12,13,14,15,16,17,18].

Globally, the proportion of late-presenting PLWH ranges between 15% and 43%, depending on the definition of late presentation definition that is used [19]. Particularly concerning figures have been reported between 2010 and 2016 in regions such as Africa (40% to 90%), Asia (72%), and Europe (48.4%) [8,20,21]. In Latin America, countries like Ecuador highlight the severity of this problem, with estimates for 2020 indicating that 29% of people living with HIV in Ecuador had late-stage infections, and 18% had advanced infections [22]. 

Many nations have recognized the dangers of late HIV diagnosis and have taken steps to address it. For example, the United States adopted universal screening in 2006, followed by the United Kingdom in 2008, France in 2009, and Spain in 2014 [23]. In contrast, Ecuador historically focused its HIV screening efforts on vulnerable groups such as pregnant women, people with sexually transmitted infections, and tuberculosis patients [24]. Additionally, HIV diagnosis and subsequent testing were largely restricted to public laboratories, often leading to significant delays in patients’ access to and management of healthcare. In response, Ecuador renewed its approach in 2018 with the “National Multisector Strategic Plan for Response to HIV/AIDS and STIs 2018–2022”, introducing universal screening under an opt-out model. This strategy involved offering HIV testing routinely—especially to the sexually active population—unless individuals explicitly declined, and it was supported by the allocation of necessary resources to reinforce its implementation [25]. This initiative aimed to improve patient care standards by ensuring rapid infection detection and efficient system linkage and promoting community-based screening campaigns. It emphasized sexual education and physician training for pre- and post-test counseling, designed to reduce underdiagnosis, prevent patient dropout, and promptly start ART, thereby mitigating health and economic impacts [25]. 

However, despite these proactive steps, there is a notable absence of empirical research evaluating the effectiveness of these strategies in Ecuador. Specifically, no study has delved into the complexities of late HIV diagnosis or measured the impact of the 2018_2022 strategic plan. Thus, the objective of this study is to describe PLWH with late and advanced presentations of HIV and evaluate the effectiveness of the strategic plan, using data from a tertiary referral hospital in Quito, Ecuador.

## 2. Materials and Methods

### 2.1. Study Design 

We undertook a cross-sectional, observational study by examining the clinical records at the “Hospital de Especialidades Eugenio Espejo” located in Quito. This institution stands as the largest specialized public hospital in the city.

### 2.2. Settings and Population 

Ecuador, nestled on the equatorial line, graces the eastern seaboard of South America. Spanning an area of approximately 283,560 km^2^, it is geographically segmented into four distinct regions: the Coast, the Andean Highlands, Amazonia, and the Galapagos Islands. From a governance perspective, Ecuador is administratively divided into 24 provinces [26].

This investigation encompassed the medical records of PLWH newly diagnosed with HIV at the Comprehensive Care Unit dedicated to individuals living with HIV in a tertiary public hospital situated in Quito, Ecuador. The hospital, while serving as a major specialized public institution, is not specifically designated as a national referral center for complicated or late-presenting HIV cases. We assessed data across two distinct phases: the first spanning from November 2015 to December 2017 and the subsequent period from January 2018 to February 2020.

### 2.3. Sample

We obtained our PLWH data from consecutive entries in clinical records through non-probabilistic sampling. This included patients who were treated at the Hospital de Especialidades Eugenio Espejo HIV Unit during the designated study intervals and who fulfilled our established selection criteria.

### 2.4. Inclusion and Exclusion Criteria

The inclusion criteria were anchored on the medical records of patients aged over 18 years who were newly diagnosed with HIV between November 2015 and February 2020 and were treated at the Comprehensive Care Unit of the tertiary public hospital in Quito. The exclusion criteria, on the other hand, excluded those with an HIV diagnosis outside the study period, those without an initial CD4 T count in their clinical records, and those with a gap exceeding 91 days between HIV diagnosis confirmation and the first CD4 T count registration.

The primary objective of this study was to evaluate changes in HIV infection presentation over two distinct periods. We sought to understand variations in CD4 T count, early versus late diagnosis rates, and factors contributing to advanced disease presentations. Furthermore, we aimed to delineate the demographic characteristics and factors associated with the late presentation and advanced disease state of HIV infection.

#### 2.4.1. Exposure

The study compared two distinct time frames: the “first period” and the “second period”. Factors assessed included demographic and socio-behavioral determinants such as age, ethnicity, academic level, employment status, and sexual behavior.

#### 2.4.2. Outcome

The primary focus of the study was on the degree of HIV presentation as delineated by immune function. This is quantitatively gauged using CD4 T levels, which serve as a direct indicator of immune system health. The presentation is categorized as:

- Early diagnosis: CD4 T count > 350 cells/mm^3^;
- Late diagnosis: CD4 T count < 350 to 200 cells/mm^3^;
- Advanced disease: CD4 T count < 200 cells/mm^3^ or the presence of an AIDS-defining illness.

### 2.5. Sample and Data Collection

For our data collection, upon obtaining ethical approval, we accessed the “Hosvital-HIS” digital system at the Comprehensive Care Unit. This access revealed 697 medical records of patients newly diagnosed with HIV within the study period. After applying inclusion and exclusion criteria, particularly those related to with the first CD4 T count taken more than 91 days after diagnosis, a total of 107 records were discarded, leaving a preliminary count of 590. Subsequently, a meticulous review for structural or systematic errors and contradictions by two independent investigators, who evaluated each case individually, further refined this count to 436 valid medical records for the scope of this study.

To gauge the impact of Ecuador’s 2018 “Multisectoral Strategic Plan”, the patient records were divided into two groups, each spanning a duration of 25 months. The first group, labeled as “pre-plan” comprised 220 records or 50.5% of the total. The second group, labeled as “post-plan”, included 216 records or 49.5% of the total.

From these records, a multitude of demographic data was gathered. These data covered facets like sex, age, ethnicity, marital status, type of residence (either urban or rural), educational background, current employment status, immigration status, and health insurance affiliation. Additionally, to paint a clearer picture of the patients, sexual characteristics and behaviors were profiled, capturing details on sexual orientation (categorized as heterosexual, homosexual, or bisexual), behavioral categorization (distinguishing men who have sex with men with a simple yes or no), lifetime count of sexual partners, condom usage habits, and trends in illicit drug consumption. The infection-related characteristics zeroed in on the initial CD4 T count and the nature of HIV infection presentation at the point of diagnosis, differentiating between late diagnosis (characterized by CD4 T counts count between 200 and <350 cells/mm^3^) and advanced disease (characterized by CD4 T counts below 200 cells/mm^3^ or the presence of an AIDS-defining illness).

### 2.6. Statistical Analysis

Categorical variables were analyzed descriptively using frequencies and percentages, while quantitative variables were evaluated using the mean and standard deviation. The chi-square test was used to determine differences between categorical variables in the first period versus the second period, while Student’s *t*-test was used to compare the means of quantitative variables. A logistic regression model was used to evaluate the association between the characteristics of individuals with late or advanced HIV presentation in the two periods studied. The results were expressed as odds ratios (OR) and their 95% confidence intervals (95% CI). A *p*-value of less than 0.05 was considered to indicate statistical significance.

## 3. Results

### 3.1. Demographic Characteristics

Of the total 436 records from both periods, the vast majority were male, comprising 392 patients (89.9%). Similarly, most patients were under 30 years of age (*n* = 236; 54.1%), 335 (95.7%) identified as of mestizo ethnicity, and 249 (87.4%) resided in urban areas. Regarding sexual orientation, the majority identified as homosexual or bisexual, comprising 295 patients (69.1%). Among the male cohort, 93 individuals (23.7%) identified as “men who have sex with men” (MSM) (Table 1).

### 3.2. Type of Disease Presentation 

Regarding the type of disease presentation, the majority of patients 65.1% (*n* = 284) were classified in the late presentation group. There was a marginal increase in the number of patients during the second period (January 2018–February 2020), but this change was not statistically significant. While 152 patients (34.9%) presented with an advanced stage of the disease, the frequency of advanced presentations decreased in the second period (*p* = 0.387). Furthermore, the mean CD4 T count was higher in the second period, although the difference was not statistically significant (*p* > 0.05) (Table 2). 

### 3.3. Associated Factors of Late Diagnosis and Advanced Infection

Throughout the sample, the distribution frequencies between PLWH with late and advanced HIV diagnoses were homogeneous according to demographic characteristics and sexual behaviors, such as sexual preference, condom use, number of partners, and even the variable “men who have sex with men”, which evaluated only the group of men. This distribution indicates a higher proportion of late presenters across all studied subgroups. Consequently, none of the participant characteristics were significantly associated with advanced HIV diagnosis (Table 1). 

### 3.4. Differences in Presentation Between First and Second Period Participants

This study assessed differences in the clinical presentation of patients at the time of HIV diagnosis between the two periods. Although no direct process indicators (e.g., screening coverage rates) were available, the analysis relied on a historical control design, comparing proportions of late and advanced presentations before and after the implementation period. Differences were observed across demographic and behavioral variables. Among those in the second period, a more significant portion of employed patients (*n* = 98 (57.3%)) presented late (*p* = 0.009), and a similar trend was found among those reporting drug use (*p* < 0.001) (Table 3). For PLWH with advanced disease presentation, the second period saw the largest group of late presenters comprising employed patients (*n* = 56 (57.7%) (*p* < 0.001)), migrants (*n* = 21 (61.8%) (*p* = 0.045)), and patients who used drugs (*n* = 32 (64.0%) (*p* = 0.001)) (Table 3).

## 4. Discussion

This study represents a crucial initial step in characterizing HIV patient diagnoses in Ecuador. Additionally, we assessed the implementation of a screening strategy promoted by the Ministry of Health of Ecuador, as outlined in the updated Multisectoral Strategic Plan. To conduct this study, we relied on data from a specialized unit for HIV patients, specifically the Unit for Comprehensive Care of People with HIV, located in a third-level public hospital that serves as a national reference for HIV diagnosis. The goal was to obtain representative results applicable to the entire Ecuadorian population.

Globally, there has been a notable decline in new HIV diagnoses over the past decade, from 2.1 million in 2011 to 1.3 million in 2022 [27,28]. However, late presentation of the disease remains a significant issue, negatively impacting the health of individuals with HIV. In our research, we found that most cases (65.1%) constituted late diagnoses, a higher proportion compared to earlier studies in various nations within the region, such as Mexico (61%) and Brazil (44%) [29,30]. It is important to note that these studies analyzed data from the early 2000s and focused on late HAART initiation, defined as a CD4 count ≤200 cells/mm^3^ combined with a treatment delay of more than six months from diagnosis. In contrast, our findings also differ from more recent international studies using standardized definitions. For example, in China (2008–2020), 55.1% of cases met the criteria for late presentation [31] while in Sierra Leone, 75.4% of patients presented at a late stage in 2017 [32]. These studies define late presentation as a CD4 count <350 to 200 cells/mm^3^ and advanced disease as a CD4 count <200 cells/mm^3^ or an AIDS-defining illness—criteria that we also adopted to ensure comparability, highlighting the complex variability in the proportion of late diagnoses worldwide. Of particular concern is the increase in the proportion of late diagnoses observed between the first (2015–2017) and second (2018–2020) study periods (*p* = 0.387), a trend also observed in some regions of the world like South Africa and England, where the proportion of late diagnoses increased from 35% in 2016 to 42% in 2021 [27].

These findings suggest that, despite the implementation of health policies promoting universal HIV screening in Ecuador, the proportion of late diagnoses increased. Although not statistically significant, this trend raises important questions about the underlying factors. However, it is crucial to note that an increase in late presenters does not necessarily reflect poor performance of the Multisectoral Strategic Plan. On the contrary, the plan may have led to broader testing coverage, thereby identifying individuals who previously would have remained undiagnosed until even later stages. This phenomenon has been reported in other settings following the introduction of routine or opt-out testing. It is important to acknowledge that the period evaluated (2018–2020) may be too short to fully capture the long-term effects of the Multisectoral Strategic Plan launched in 2018. Policy impacts, especially those related to public health education, system-wide implementation, and behavior change, often require more time to be reflected at the population level. Therefore, our results should be interpreted as an early evaluation, underscoring the need for ongoing monitoring and future follow-up studies to more thoroughly assess the plan’s effectiveness. 

Although our study did not directly investigate structural or sociocultural barriers to HIV testing, the patterns observed—particularly the high proportion of late and advanced diagnoses among migrants, individuals who use drugs, and employed patients—may reflect systemic challenges in accessing timely testing and care. These groups often face hidden or underexplored barriers, including stigma, fear of discrimination, legal insecurity, inflexible work schedules, or lack of outreach services. Therefore, strategies may require refinement, including adequate allocation of financial resources to promote widespread testing, maintaining a stable supply of HIV-related materials, providing ongoing training to healthcare personnel, and expanding testing options beyond traditional healthcare settings. These adjustments could help reach populations that are less likely to access routine screening, as suggested by the distribution patterns observed in our cohort.

Regarding the population analyzed in this study, most patients were male (89.9%), urban residents (87.4%), lacking health insurance (95.6%), and identifying as homosexual or bisexual (69.1%). These findings align with previous research identifying a higher frequency of late diagnosis among men, adults, and individuals with homosexual or bisexual orientations [33,34,35]. On the other hand, although various factors—such as advanced age have previously been associated with late and advanced diagnosis primarily due to the aging of the immune system, low educational level as a trigger for low socioeconomic conditions and limited access to healthcare, or sexual orientation among homosexuals and men who have sex with men [17,29,34,36,37,38,39]—our overall risk analysis, including the two periods for advanced diagnosis, did not identify any associated characteristics between demographic variables and sexual characteristics.

However, some differences emerged when analyzing late and advanced presentation by period. Notably, there was a rise in the proportion of patients who reported drug use in the second period. While our study does not directly evaluate national drug policy, this trend may be partially explained by broader social and legal developments in Ecuador. In 2013, Ecuador reformed its criminal code to decriminalize the possession of small quantities of illicit drugs for personal use, aiming to reduce punitive enforcement and promote public health approaches. Additionally, discussions around harm reduction—including pilot substitution therapies and treatment services—gained visibility during the late 2010s. These policy shifts may have contributed to increased self-reporting or service access among people who use drugs during the second period of our study [40,41]. 

Concerning advanced diagnoses, we also observed a significant increase in migrant patients during the second period. This may be explained by increased migration flows into Ecuador from countries experiencing socioeconomic or political instability. Migrant populations often face multiple barriers to healthcare, including lack of legal status, unfamiliarity with the healthcare system, language barriers, and discrimination [42]. These challenges can delay health-seeking behaviors and contribute to more advanced disease at diagnosis. Additionally, fear of deportation and job insecurity may discourage migrants from accessing HIV screening and care services. 

The higher proportion of late and advanced diagnoses among people who use drugs—particularly during the second period—likely reflects the combined impact of increased transmission risk and barriers to healthcare access. Individuals who use drugs often encounter complex structural challenges such as stigma, criminalization, and marginalization, which are widely recognized as factors contributing to reduced testing uptake and delayed linkage to care. Our findings align with global evidence indicating that substance use is associated with late HIV diagnosis and lower engagement in care [43]. 

In both study periods, the percentage of employed individuals with late or advanced diagnoses was notably high. This may be due to limited healthcare access caused by inflexible work schedules, lack of employer-based screening programs, or stigma associated with disclosing HIV status in the workplace [44]. Additionally, the higher proportion of unemployed individuals with late diagnoses in the first period may reflect the economic context of the time, in which socioeconomic vulnerability and lack of insurance may have limited access to timely testing and care.

Regarding condom use, although no statistically significant association with late diagnosis was found, patients who reported never or irregular condom use consistently showed higher rates of late or advanced presentation. This points to shortcomings in HIV prevention strategies, particularly in sexual education and condom promotion. These findings support the need to integrate behavioral risk assessment and harm reduction approaches into screening efforts, especially for individuals with inconsistent preventive practices.

HIV screening is a widely used tool worldwide to achieve early diagnosis of HIV infection. In Ecuador, following the implementation of the 2018–2022 Multisectoral Strategic Plan, the Ministry of Public Health adopted an opt-out screening approach. Testing is offered primarily through public healthcare facilities, where it is free of charge and supported by national guidelines. However, access remains limited in rural or underserved areas, and out-of-pocket costs may still arise in private sector settings or when confirmatory testing is performed outside the public network [25]. This study sheds light on the implementation of HIV screening in Ecuador and the remaining barriers to early detection of the disease.

To improve early diagnosis and mitigate the impact of HIV in Ecuador, public health policies must incorporate several key strategies. One such strategy is implementing point-of-care testing, which provides results in minutes using finger-prick blood or saliva samples. This approach is particularly beneficial in settings where patients face difficulties attending follow-up medical appointments or when rapid diagnosis is crucial for immediate clinical treatment. Widely used globally and first approved by Health Canada in 2005, point-of-care HIV testing offers high sensitivity and specificity, although it must be followed by laboratory testing for confirmation [45].

Additionally, access to HIV testing in Ecuador requires addressing the challenges posed by the country’s divided public and private healthcare system and ensuring testing is accessible and affordable is essential, especially given the high cost of drugs and the difficulties in negotiating lower prices due to the country’s small market size. Public health policies should focus on increasing early diagnosis in high-risk areas and among vulnerable populations, such as sex workers, men who have sex with men, and transgender people. This can significantly reduce the spread of HIV. By prioritizing these strategies, Ecuador can improve its response to HIV and enhance the health outcomes of its population.

### Limitations

This study is subject to several limitations, notably information and selection biases. Information bias arises due to data collection from medical records, which constrained the availability of additional variables essential for a comprehensive characterization of the studied population. For instance, medical records lacked information on whether the values represented the initial test in the HIV diagnosis process, whether it was requested or offered by healthcare personnel, or if it was a voluntary request due to individual risk perceived by the patient. This limitation prevented an isolated evaluation of each strategic line proposed in the Multisectoral Plan. Additionally, health professionals who compiled data from medical records did not differentiate between homosexual orientation and MSM status, causing overlap in the results.

Moreover, the study’s design precluded the use of sampling methods that would have facilitated result generalization to the entire population. Despite these constraints, the results remain significant, as they derive from patients treated at a specialized HIV unit within a referral hospital in Ecuador, serving as a vital resource for HIV diagnosis. Furthermore, our findings identify patterns and associations that are consistent with prior research across different regions and time periods. Notably, this study represents one of the first efforts to characterize late and advanced HIV disease presentation in Ecuador. While these results are relevant and provide valuable insights into a previously understudied population, our study does not clarify the effect of the Multisectoral Strategic Plan on its primary objective—reducing the proportion of individuals diagnosed with late or advanced HIV disease. First, other studies in different settings may yield divergent findings. Second, the plan’s effect—if any—could be either beneficial or detrimental, depending on implementation quality and context. As such, these results should be interpreted as a preliminary contribution that highlights the need for further research and systematic evaluation of policy outcomes.

## 5. Conclusions

In this study evaluating the presentation of HIV disease, it was identified that the majority of patients (65.1%) were diagnosed at a late stage of the disease. Among these patients, the rate increased in the post-plan period, although advanced disease diagnoses were less frequent in the post-plan period. Notably, demographic characteristics did not show a clear influence on the advanced presentation of HIV infection. However, differences between the two periods revealed that, in the post-plan period, employed patients and those who reported drug use were more likely to present late, and the latter also accounted for a significant proportion of advanced disease presentations. Despite these differences, it appears that the implementation of the HIV/AIDS and STI response plan has not had a clear effect on reducing late and advanced HIV disease among Ecuadorians.

## Figures and Tables

**Table 1:** Demographic characteristics of patients and risk of presentation (late and advanced) according to population characteristics.

		Total	Late Diagnosis		Advanced Disease		OR (95%CI)
		*n*	*n*	%	*n*	%	
Sex	Female ref.	44	29	65.9	15	34.1	
	Male	392	255	65.1	137	34.9	1.038 (0.538–2.003)
Age (years)	<30 ref.	236	157	66.5	79	33.5	
	>30	200	127	63.5	73	36.5	1.142 (0.769–1.695)
Ethnicity	No Mestizo ref.	15	11	73.3	4	26.7	
	Mestizo	335	213	63.6	122	36.4	1.575 (0.490–5.053)
	N/A	86					
Marital status	Single/Divorced ref.	386	251	65.0	135	35.0	
	Married/Free union	50	33	66.0	17	34.0	0.957 (0.514–1.783)
Residential area	Urban ref.	381	249	65.4	132	34.6	
	Rural	55	35	63.6	20	36.4	1.077 (0.598–1.941)
Academic Level	>12 years ref.	318	209	65.7	109	34.3	
	<12 years	113	71	62.8	42	37.2	1.134 (0.725–1.772)
	N/A	5					
Employment status	Unemployed ref.	165	111	67.3	54	32.7	
	Employee	268	171	63.8	97	36.2	1.166 (0.774–1.756)
	N/A	3					
Immigration status	No migrant ref.	329	211	52.9	118	47.1	
	Migrant	107	73	68.2	34	31.8	0.832 (0.523–1.326)
Health insurance	Affiliate ref.	19	12	63.2	7	36.8	
	Unaffiliated	417	272	65.2	145	34.8	0.913 (0.352–2.371)
Sexual preference	Heterosexual ref.	132	81	61.4	51	38.6	
	Homosexual/bisexual	295	196	66.4	99	33.6	0.802 (0.524–1.227)
	N/A	9					
Men who have sex with men	Yes ref.	93	55	59.1	38	40.9	
	No	291	194	66.7	97	33.3	0.723 (0.447–1.169)
	N/A	8					
Sexual partners	>10 ref.	129	76	58.9	53	41.1	
	<10	181	125	69.1	56	30.9	0.642 (0.400–1.029)
	N/A	126					
Condom use	Regular ref.	125	83	66.4	42	33.6	
	Never/irregular	194	122	62.9	72	37.1	1.166 (0.727–1.869)
	N/A	117					
Drug use	No ref.	268	173	64.6	95	35.4	
	Yes	147	97	66.0	50	34.0	0.938 (0.614–1.433)
	N/A	21					

N/A, not available.

**Table 2:** Characterization of patients’ HIV infection presentation.

		First Period		Second Period		Total	*p*-Value
		*n*	%	*n*	%		
Presentation of HIV infection	Late (CD4 T < 350 cells/mm^3^)	139	48.9	145	51.1	284	0.387
	Advanced disease (CD4 T < 200 cell/mm^3^)	81	53.3	71	46.7	152	
		Mean	±(SD)	Mean	±(SD)		
CD4 T count (cells/mm^3^)		284	±206.9	308	±195.0		0.284

**Table 3:** Differences between participants in the first and second periods, according to the presentation of infection (late and advanced).

		Total	Late Diagnosis					Advanced Disease					
			First Period		Second Period		*p*-Value	Total	First Period		Second Period		*p*-Value
		*n*	*n*	%	*n*	%		*n*	*n*	%	*n*	%	
Sex	Female	29	17	58.6	12	41.4	0.271	15	10	66.7	5	33.3	0.274
	Male	255	122	47.8	133	52.2		137	71	51.8	66	48.2	
Age (years)	<30	157	79	50.3	78	49.7	0.606	79	39	49.4	40	50.6	0.313
	>30	127	60	47.2	67	52.8		73	42	57.5	31	42.5	
Ethnicity	No Mestizo	11	4	36.4	7	63.6	0.385	4	2	50.0	2	50.0	0.845
	Mestizo	213	106	49.8	107	50.2		122	67	54.9	55	45.1	
Marital status	Single/Divorced	251	127	50.6	124	49.4	0.124	135	75	55.6	60	44.4	0.114
	Married/Free union	33	12	36.4	21	63.6		17	6	35.3	11	64.7	
Residential area	Urban	249	120	48.2	129	51.8	0.499	132	69	52.3	63	47.7	0.518
	Rural	35	19	54.3	16	45.7		20	12	60.0	8	40.0	
Academic level	>12 years	209	99	47.4	110	52.6	0.192	109	55	50.5	54	49.5	0.206
	<12 years	71	40	56.3	31	43.7		42	26	61.9	16	38.1	
Employment status	Unemployed	111	65	58.6	46	41.4	0.009	54	39	72.2	15	27.8	<0.001
	Employee	171	73	42.7	98	57.3		97	41	42.3	56	57.7	
Immigration status	No migrant	211	108	51.2	103	48.8	0.199	118	68	57.6	50	42.4	0.045
	Migrant	73	31	42.5	42	57.5		34	13	38.2	21	61.8	
Health insurance	Affiliate	12	4	33.3	8	66.7	0.269	7	2	28.6	5	71.4	0.179
	Unaffiliated	272	135	49.6	137	50.4		145	79	54.5	66	45.5	
Sexual preference	Heterosexual	81	40	49.4	41	50.6	0.951	51	27	52.9	24	47.1	0.944
	Homosexual/bisexual	196	96	49.0	100	51.0		99	53	53.5	46	46.5	
Men who have sex with men	No	55	26	47.3	29	52.7	0.877	38	19	50.0	19	50.0	0.787
	Yes	194	94	48.5	100	51.5		97	51	52.6	46	47.4	
Sexual partners	>10	125	71	56.8	54	43.2	0.101	56	33	58.9	23	41.1	0.162
	<10	76	52	68.4	24	31.6		53	38	71.7	15	28.3	
Condom use	Regular	83	35	42.2	48	57.8	0.147	42	15	35.7	27	64.3	0.028
	Never/irregular	122	64	52.5	58	47.5		72	41	56.9	31	43.1	
Drug use	No	173	100	57.8	73	42.2	< 0.001	95	60	63.2	35	36.8	0.001
	Yes	97	35	36.1	62	63.9		50	18	36.0	32	64.0	

## Data Availability

The original contributions presented in this study are included in the article. Further inquiries can be directed to the corresponding author.
